# Supplementary material for: Inflammatory Intracellular Signaling in Neurons Is Influenced by Glial Soluble Factors in iPSC-Based Cell Model of PARK2-Associated Parkinson’s Disease
Source: Int J Mol Sci. 2024 Sep 5;25(17):9621. doi: 10.3390/ijms25179621 (PMC11395490; doi:10.3390/ijms25179621)
Supplement: Supplementary file 1 [file ijms-25-09621-s001.zip › Supplementary Figure S1-S5pdf.pdf]

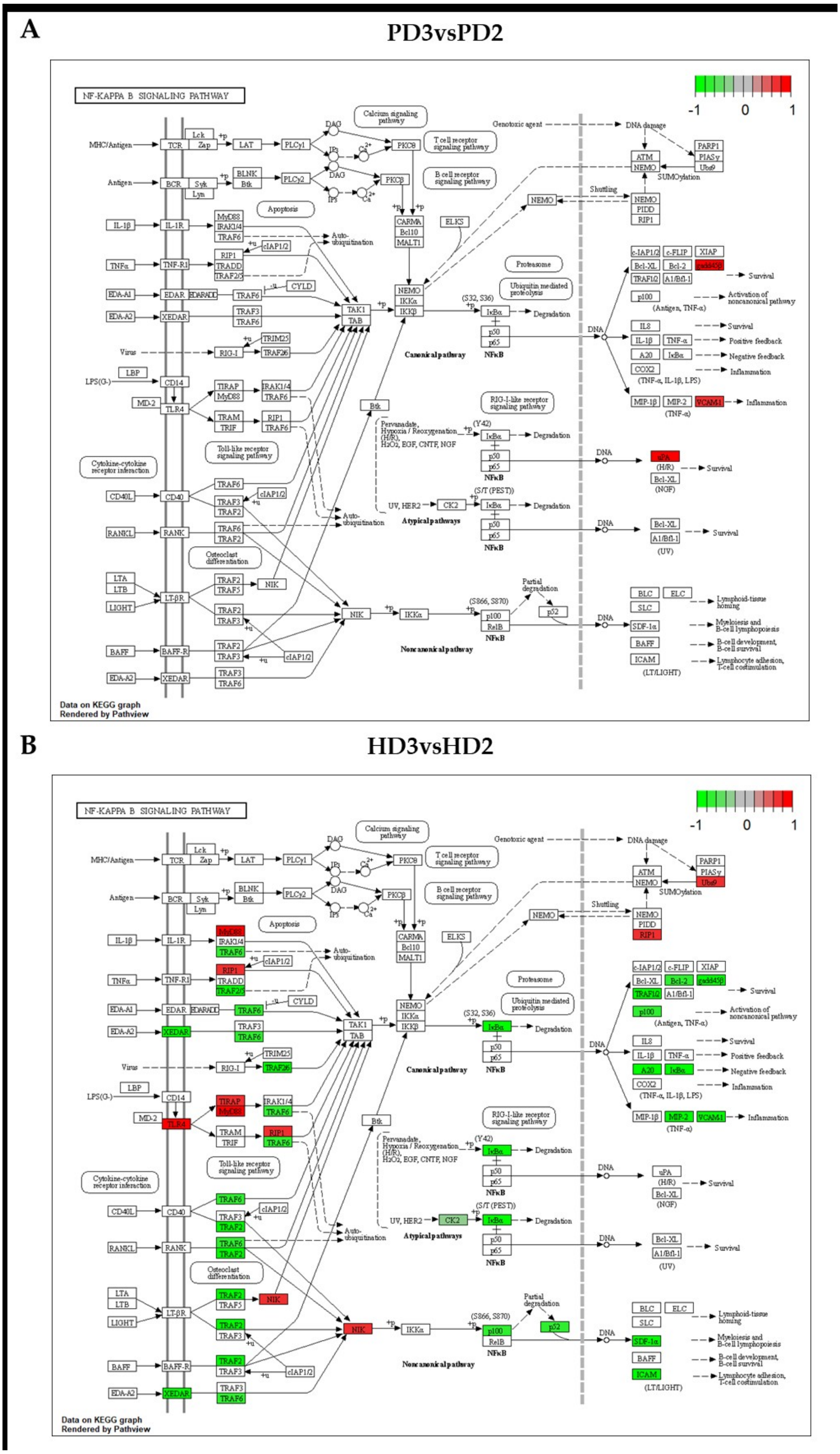

Figure S1. NF-kB signaling. KEGG graphs. (**A**) Comparison between PD neurons in PD and HD glial mixed medium. (**B**) Comparison between HD neurons in PD and HD glial mixed media. All the genes are presented, only DEGs are given in colors: green—downregulated, red—upregulated genes.

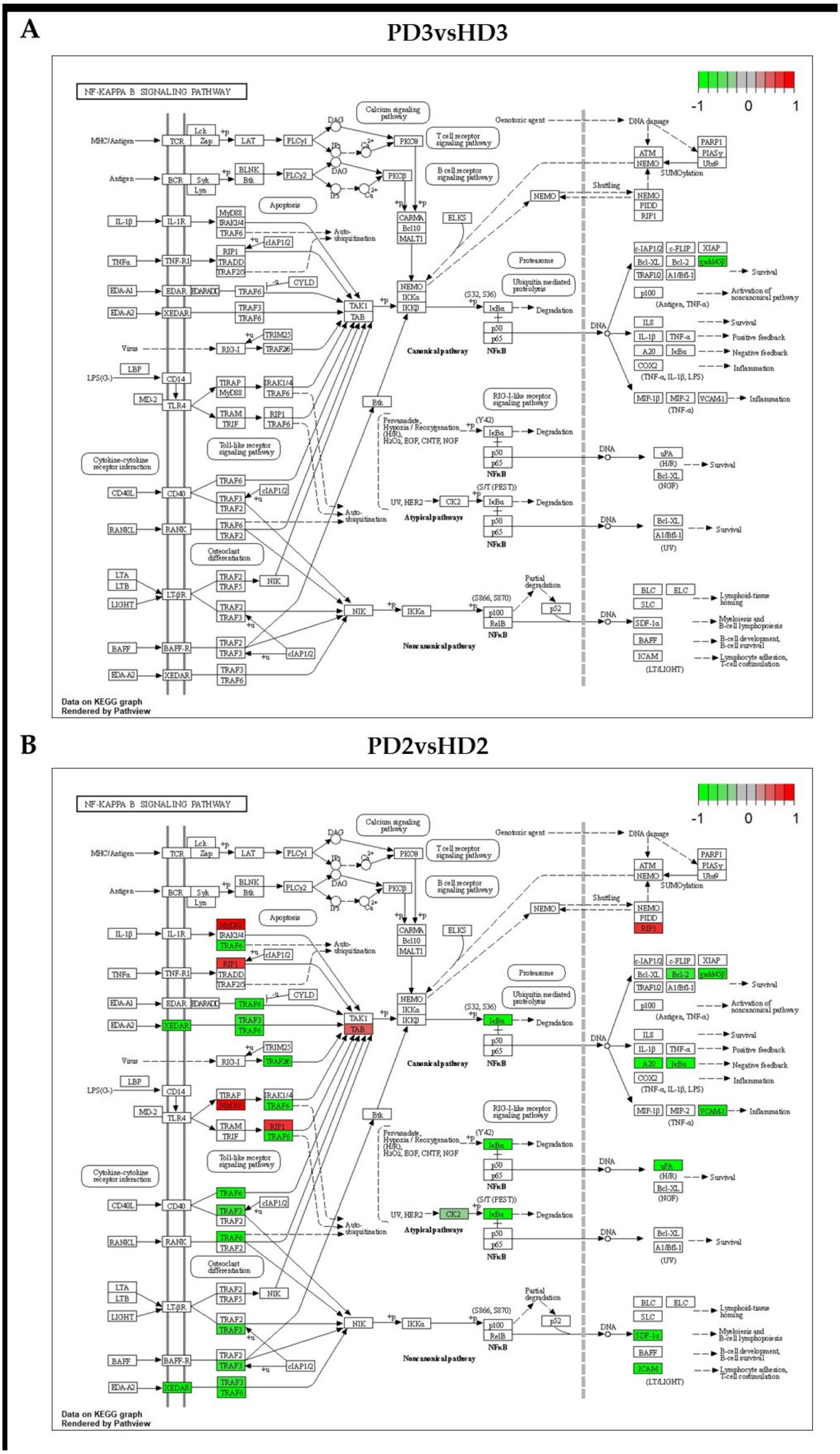

Figure S2. NF-kB signaling. KEGG graphs. (**A**) Comparison between PD and HD neurons in PD glial mixed medium. (**B**) Comparison between PD and HD neurons in HD glial mixed medium. All the genes are presented, only DEGs are given in colors: green—down-regulated, red—upregulated genes.

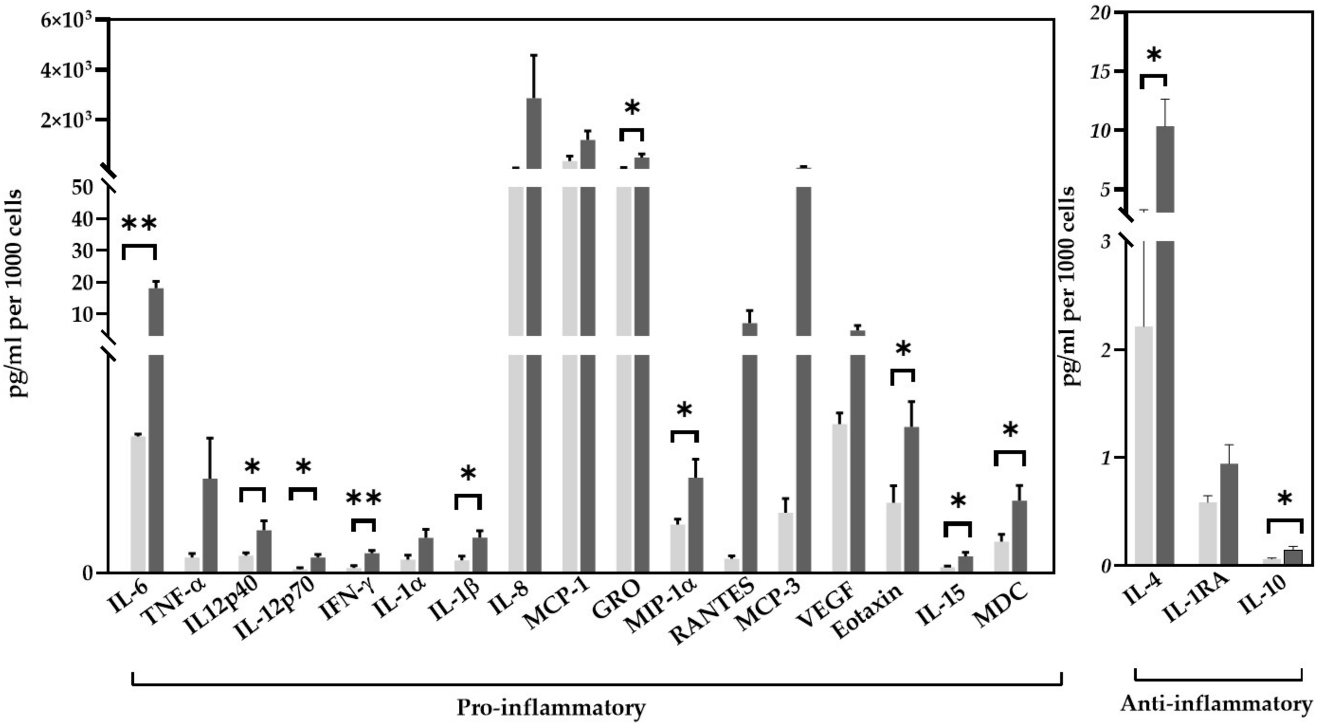

Figure S3. Cytokine/chemokine composition of HD and PD individual glial supernatants. Multiplex analysis. Light-gray columns—concentrations in HD glial supernatants, dark-gray columns—concentrations in PD glial supernatants. The results are presented as mean ± SEM (samples were taken in two biological replicates). Recalculated per 1000 cells. Statistical analysis was performed using unpaired two-tailed t-test (GraphPad Prism 8.0.1. software). The differences were considered statistically significant at * $p < 0.05$, ** $p < 0.01$.

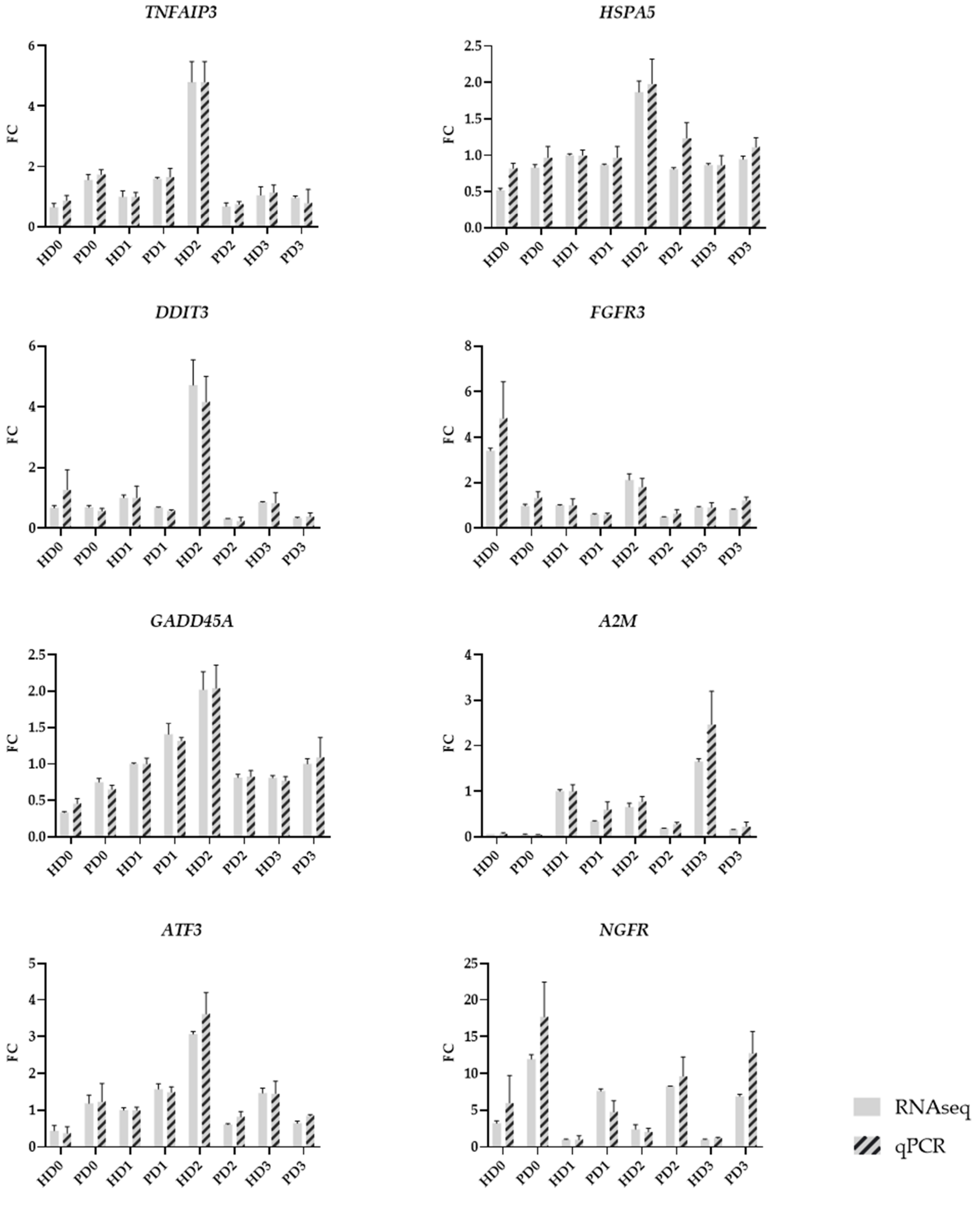

Figure S4. The expression of key DEGs in HD and PD (*hom EX8 del PARK2*) neurons cultivated in different types of media. RNAseq data and qPCR results were normalized to the mean level of gene expression in HD1. No shading—RNAseq data; shaded—qPCR validation. 0—base neuronal medium, 1—base glial medium, 2—HD glial mixed medium, 3—PD glial mixed medium.

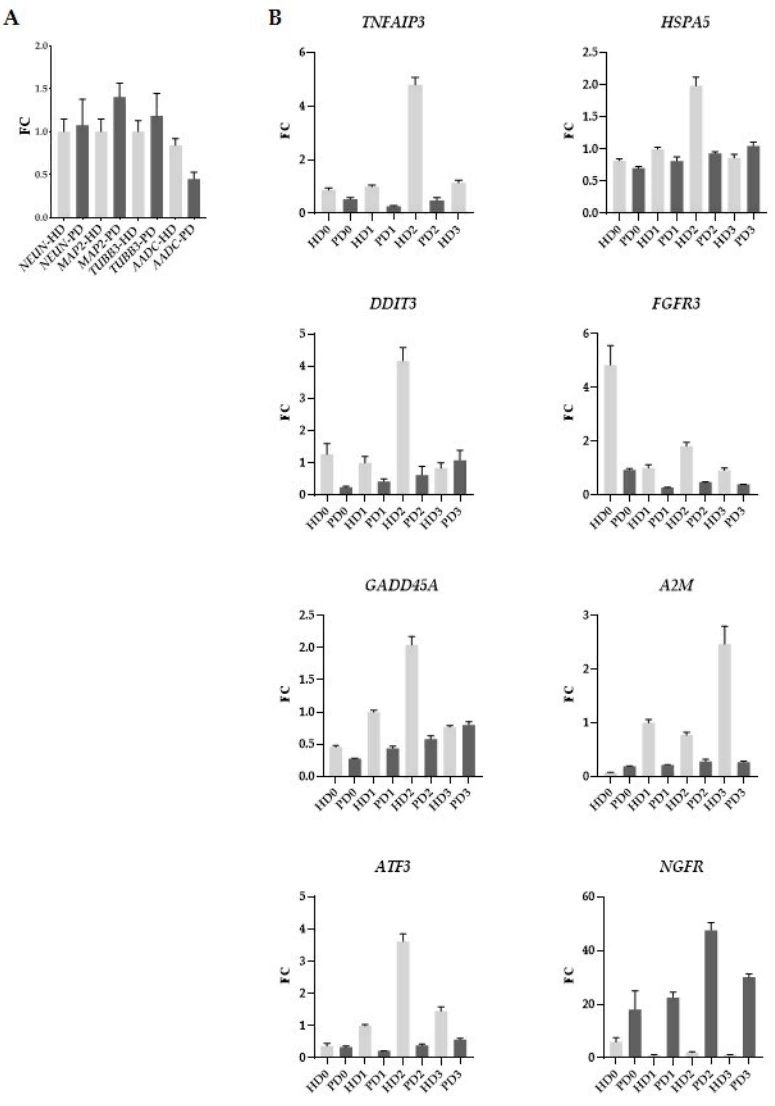

Figure S5. qPCR data on the expression of key DEGs in the additional PD neuronal culture (*het EX2 del PARK2*). (**A**) qPCR data on mature neurons' markers normalized to the mean level of gene expression in HD. The differences were considered statistically non-significant at $p > 0.05$; (**B**) qPCR data on DEGs expression normalized to the mean level of gene expression in HD1. Light-gray—HD neurons, dark-gray—PD neurons; 0—base neuronal medium, 2—HD glial mixed medium, 3—PD glial mixed medium.
